# Hyperbaric oxygen treatment and nephrotoxicity induced by gentamicin in rats

**DOI:** 10.1186/s12882-017-0768-2

**Published:** 2017-12-02

**Authors:** Matitiahu Berkovitch, Yossi Shain, Eran Kozer, Michael Goldman, Ibrahim Abu-Kishk

**Affiliations:** 1 Pediatric Division, Assaf Harofeh Medical Center, 70300 Zerifin, Israel; 2 Sackler School of Medicine, Tel Aviv University, Tel Aviv, Israel

**Keywords:** Hyperbaric oxygen therapy, Gentamicin, Nephrotoxicity, Rat model

## Abstract

**Background:**

Nephrotoxicity is a significant adverse side effect of gentamicin. Previous preclinical studies showed that hyperbaric oxygen treatment (HBOT) may have beneficial effects by attenuating renal damage in rats subjected to renal injury. We evaluated the effect of HBOT on acute renal failure caused by gentamicin.

**Methods:**

Thirty-six rats were divided into four groups. Gentamicin (150 mg/kg for 5 consecutive days) was administered in 30 rats, 10 rats received only gentamicin, 10 rats received 100% oxygen therapy on days 1-5 of the experiment, 10 received daily HBOT on days 1-5 of the experiment, and the remaining six served as a control group. On day 6, renal function tests and renal pathological examinations were performed.

**Results:**

Body weight and biochemical parameters were similar in all groups except for higher plasma levels of calcium in the 100% oxygen group (*P* = 0.03). All the rats in the experimental group showed biochemical parameters compatible with renal failure (high serum levels of urea and creatinine). All the rats in the control group had normal renal function tests. Two rats from the HBOT group died on the fifth day of the experiment. All rats in the control group demonstrated normal renal morphology. All 28 intoxicated rats showed moderate to severe histopathological changes without significant differences between the groups.

**Conclusions:**

Treatment of gentamicin-induced nephrotoxicity with either HBOT or 100% oxygen for 5 days had no beneficial renal effect. Mortality was observed only in the HBOT group.

## Background

Gentamicin is an aminoglycoside antibiotic used to treat many types of bacterial infections, particularly those caused by gram-negative organisms. However, gentamicin may cause severe renal toxicity.Nephrotoxicity is a major problem in its clinical use [1]. Oxidative stress has been reported in the tubular toxicity of gentamicin and several antioxidant products have been used to protect against gentamicin-induced renal toxicity [2, 3].

Hyperbaric oxygen therapy (HBOT) - breathing 100% oxygen at a pressure above the atmospheric pressure at sea level [> 1 atm absolute (ATA), 760 mmHg] can result in increased arterial and tissue oxygen tension [4]. HBOT treatment may have beneficial effects on the kidneys. Several studies have demonstrated that HBOT treatment attenuates the increase in plasma creatinine, the deterioration in glomerular filtration rate, and reduces oxidative stress and the histopathologic damage in rats subjected to renal sepsis or ischemia/reperfusion injury [5, 6].

In a previous study conducted by our team, we showed that the use of HBOT in a rat model at similar doses used for therapeutic purposes in human subjects has no harmful renal effect [7].

The current study aimed to evaluate the biochemical and the pathological efficacy of HBOT on acute renal failure induced by gentamicin.

## Methods

### Animals

The study was approved by the Assaf Harofeh Medical Center Animal Care Committee. Male Sprague-Dawley rats, aged 6-8 weeks and weighing 220-270 g were studied. All the rats were supplied by Envigo CRS (Israel) Ltd. The rats were handled in strict adherence to the Institutional Animal Care and Use Committee (IACUC) standards. They were double-housed in shoe-box-type cages and had free access to food and water in the week prior to administration of gentamicin. Starting from 12 h prior to gentamicin administration and until gentamicin was administered, food was withheld from the rats; free access to water was given. After gentamicin administration, the rats had free access to food and water. Animal weight was measured and recorded every day during the experiment.

All procedures were performed under sedation with carbon dioxide (CO2), and all efforts were made to minimize suffering. Each animal was placed in an empty clean chamber; the flow of CO2 from the gas cylinder was started at a rate that would displace 10–30% of the chamber volume per minute. This calculated rate would allow a slow increase in the concentration of CO2 to develop, but would not cause noise or be perceived as harsh “wind” to the animals. As gas levels rose to 40–50%, unconsciousness occurred as indicated by a loss of the righting reflex. At this point, the procedures with the rats were performed. At the end of the experiment protocol, the animals were sacrificed by cervical dislocation after CO2 sedation.

The rats were monitored twice daily by a staff member authorized by the institutional veterinarian. If one of the following conditions occurred during the experiment, the animal was sacrificed and experiment termination considered, according to the decision of the institutional veterinarian: signs of suffering or pain that could not be treated with analgesics; significant changes in physiological parameters (breathing, heart rate, social behavior); other signs of distress (apathy, prolonged lying, aggressive behavior, self harm, anorexia, hyperactivity); refusal to eat or drink independently for 48 h and 24 h respectively; an infection, swelling or inflammation that could not be treated; loss of more than 10% of baseline weight.

### Gentamicin preparation and administration

Ampules of 2 ml containing 80 mg of gentamicin sulfate (Teva Pharmaceutical Works Private Limited Company, Hungary) were used. The rats were weighed on digital scales. The total dose per rat was calculated individually and the solution was injected intraperitoneally under sedation with CO2.

### Hyperbaric oxygen administration

Hyperbaric oxygen was administered using a purpose-built animal hyperbaric chamber manufactured locally by our team of medical engineers. This 20 l cylindrical chamber is supplied with 100% oxygen through a single entry and equipped with an atmospheric pressure gauge. The measured oxygen concentration within the chamber was 100%. Treatment consisted of administration of 100% oxygen at 2 ATA for 70 min, including 5 min of compression and 5 min of decompression time.

### Experimental protocol

The study protocol was divided into two phases. Phase 1 was designed to evaluate the dose of gentamicin needed for induction of renal failure. Phase 2 was designed to evaluate the effect of oxygen therapy and HBOT on rats with gentamicin-induced nephropathy.

Phase 1: In a pilot study, we found that five doses of gentamicin (150 mg/kg each) administered on five consecutive days by intraperitoneal injection induced moderate renal failure with no significant effect on mortality. Normal biochemical values were determined based on previous studies performed by our group using the same animal model and the control group in the current study [7, 8].

Phase 2: Thirty-six rats were included in the study. On day one of the experiment, 30 rats received five intraperitoneal injections of 150 mg/kg of gentamicin for five consecutive days. The rats were randomly divided into three equal groups. Group 1 received no treatment except for gentamicin administration. Group 2 received 100% oxygen therapy, started immediately after the gentamicin injection, on days 1-5 of the experiment, at a normal pressure (1 ATA) for 60 min each. Group 3 received daily HBOT started immediately after the gentamicin injection, on days 1-5 of the experiment, at a pressure of 2 ATA for 60 min each. The other six rats served as a control group. Two rats received 100% oxygen therapy only for 5 days, two rats received HBOT only for 5 days and two rats did not receive any treatment.

On day 6 of the experiment (1 day after the last dose of gentamicin), blood was drawn for biochemical evaluation of urea, creatinine and electrolytes rats by cardiac puncture. CO2 was used for sedation prior to terminal cardiac puncture.

The rats’ kidneys were immediately removed, preserved in formaldehyde and embedded in paraffin for pathological examination.

### Biochemical analysis

Evaluation of creatinine, urea and magnesium were performed using the Roche/Hitachi modular P800 autoanalyser (Roche Diagnostics, Mannheim, Germany). Evaluation of potassium was performed using the Roche/Hitachi modular ISE 900 autoanalyser (Roche Diagnostics, Mannheim, Germany). Gentamicin plasma level was performed using the Roche Cobas Integra 800 analyzer (Roche Diagnostics, Mannheim, Germany) based on fluorescence polarization, using antibody reagents and tracer reagents. In this paper, acute kidney failure was defined as doubling of creatinine levels in the plasma [8, 9]. The biochemical analyses were performed by an observer blinded to the treatment received by each animal.

### Pathological evaluation

The kidneys of each animal were dissected, fixed in buffered formalin for 12 h and processed for histopathological examination. Large sections (1 μm) were cut perpendicular to the renal capsule, in order to ensure that both cortex and medulla are presented in each section. The entire cortical areas of all the specimens were evaluated in order to assess the extent of the damage induced by the different treatment protocols. Four micrometer-thick paraffin sections were stained with hematoxylin and eosin for light microscope examination using the conventional protocol. A minimum of eight fields for each kidney section were examined and classified according to severity of changes by an observer blinded to the treatment received by each animal. The following features were monitored: tubular dilatation, intra tubular casts and interstitial inflammation. The changes were monitored using a six-tier grading system, as follows: Grade 0 –normal, grade 1 –minimal changes only (weak and focal), grade 2 - < 25% of the cortex underwent histopathological changes, grade 3–25–50%, grade 4–50–75%, and grade 5 - > 75% [8].

### Statistical analysis

The statistical analysis of the results was performed using SPSS (ver.19). The quantitative measurements are presented using mean, SD, median, min and max. One way ANOVA with post-hoc Tukey test was performed to examine whether there was a significant difference between the groups. Values were considered statistically significant when *P* < 0.05.

## Results

### Survival and body weight

All six rats in the control group survived. Table 1 presents a comparison between the groups. All the rats in the control group gained weight, in comparison to the rats in groups 1, 2,and 3 who lost weight. There were no significant differences in body weight between the intoxicated groups. All the rats in Groups 1 and 2 survived. Two rats in the HBOT group died during the HBOT treatment after the injection of the fifth dose. The death of the rats occurred suddenly during the decompression phase of HBOT. The two rats appeared cyanotic and had respiratory distress. Development of pneumothorax was the speculated etiology of this sudden event.

**Table 1:** Comparison between the groups

	Control group (*n* = 6)	Group 1 (*n* = 10)	Group 2 (*n* = 10)	Group 3 (*n* = 8)	*P*-value
Weight (gr) Day1 (±SD)	292.2 ± 12.16	289 ± 8.93 (279-304)	294.3 ± 15.37 (267-315)	299.9 ± 7.50 (289-313)	0.11
Weight(gr) Day 6 (±SD)	310.3 ± 10.31	280.8 ± 11.25 (262-297)	292.2 ± 13.39 (271-318)	287.04 ± 7.4 (280-300)	0.89
Urea (mg/dl) (±SD)	42 ± 2	218.96 ± 95.65 (93-329.6)	202.35 ± 66.08 (82.7-311.5)	229.18 ± 48.05 (170.5-298.1)	0.741
Creatinine (mg/dl) (±SD)	0.41 ± 0.02	3.43 ± 1.517 (1.58-5.58)	3.16 ± 1.162 (1.21-4.46)	3.5 ± 0.66 (2.58-4.63)	0.813
Sodium (mmol/l) (±SD)	142 ± 1.5	141.4 ± 3.23 (136-146)	140.5 ± 3.44 (132-144)	140.88 ± 2.41 (136-144)	0.811
Potassium (mmol/l) (±SD)	5.5 ± 0.3	4.62 ± 0.39 (4.06-5.8)	4.78 ± 0.36 (4.17-5.4)	4.83 ± 0.49 (4.42-5.93)	0.519
Chloride (mmol/l) (±SD)	94.2 ± 3.7	94.7 ± 3.88 (88-100)	95.4 ± 4.9 (85-103)	95.75 ± 1.98 (93-99)	0.842
Magnesium (mg/dl) (±SD)	4.13 ± 0.33	5.03 ± 0.64 (3.9-6.09)	5.3 ± 1.21 (3.58-7.23)	5.34 ± 0.52 (4.57-6.12)	0.701
Calcium (mg/dl) (±SD)	11.8 ± 0.1	11.95 ± 7.44 (11.2-13.8)	12.8 ± 0.87 (11.8-14.9)	12.06 ± 0.47 (11.2-12.6)	0.034
Gentamicin levels (μg/ml) (±SD)	0	24.24 ± 31.06 (5.2-88.4)	24.03 ± 20.15 (3.1-61.5)	30.62 ± 17.71 (9.9-64.9)	0.806

The *P*-value refers to compare between groups 1, 2, 3

Group 1 (gentamicin), Group 2 (100% oxygen therapy + gentamicin), Group 3 (hyperbaric oxygen treatment + gentamicin)

### Biochemical parameters

Biochemical values of the control group were as followed: urea (mg/dl) 40-44, creatinine (mg/dl) 0.39-0.43, calcium (mg/dl) 11.7-11.9, sodium (mmol/L) 141-144, potassium (mmol/L) 5.1-5.8 (Table 1). All the rats in groups 1, 2, and 3 developed renal failure with creatinine and urea values higher more than twofold in comparison to the control group. Table 1 presents a comparison between the groups intoxicated with gentamicin. There were no significant differences in biochemical parameters between the groups except for plasma calcium levels between Group 2 and the other groups. Group 2 had slightly elevated levels of calcium plasma levels.

### Pathologic evaluation

**Fig. 1:**
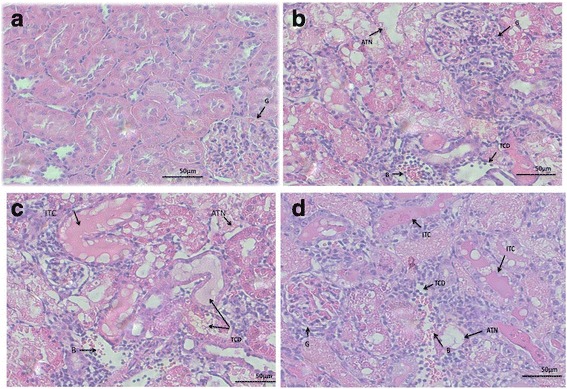
**a** Control group (kidney of rats that received no treatment or 100% oxygen or HBOT). The figure shows normal kidney, the *arrow* indicates normal glomerulus (G). **b** Group 1, (kidney of rats treated with gemtamicin alone). The *arrows* indicate acute tubular necrosis (ATN), tubular cell degeneration (TCD), bleeding (B) and normal glomerulus (G). **c** Group 2, (kidney of rats treated with gemtamicin and 100% oxygen). The *arrows* indicate acute tubular necrosis (ATN), tubular cell degeneration (TCD), intratubular casts (ITC), bleeding (B) and normal glomerulus (G). **d** Group 2, (kidney of rats treated with gemtamicin and HBOT). The *arrows* indicate acute tubular necrosis (ATN), tubular cell degeneration (TCD), bleeding (B) and normal glomerulus (G)

**Fig. 2:**
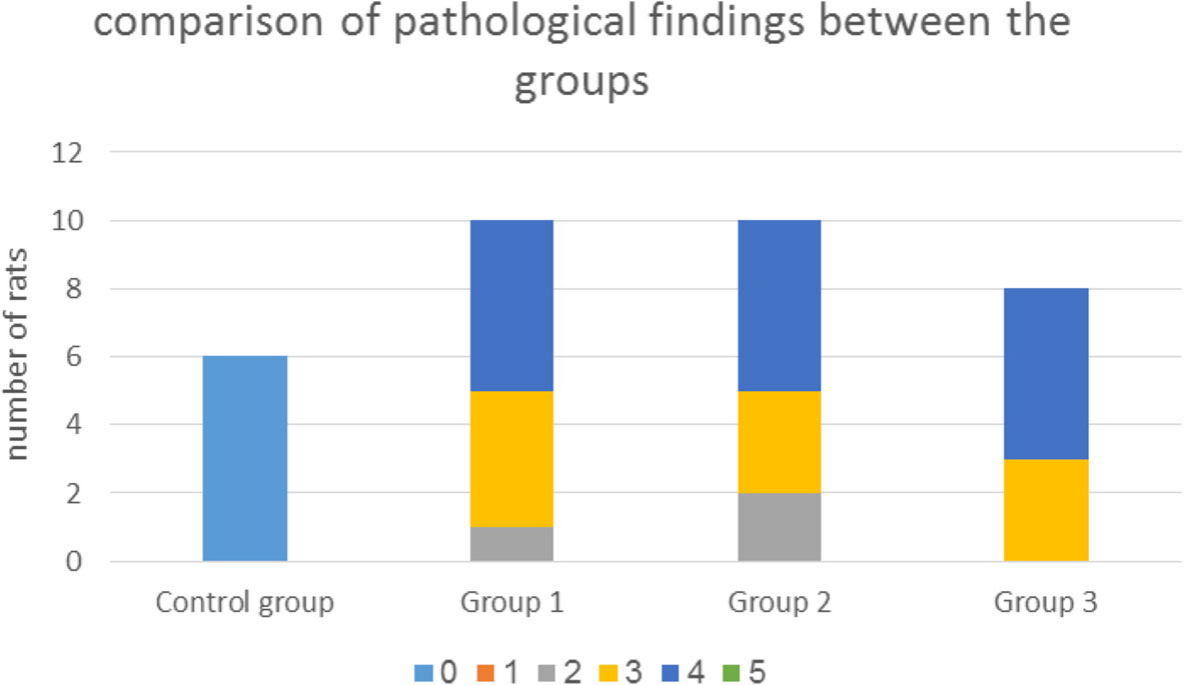
Comparison of pathological findings between the groups. Pathological score: Grade 0 –normal, grade 1 –minimal changes only (weak and focal), grade 2 - < 25% of the cortex underwent histopathological changes, grade 3–25–50%, grade 4–50–75%, and grade 5 - > 75%. Group 1 received no treatment except for gentamicin. Group 2 received 100% oxygen therapy + gentamicin. Group 3 received hyperbaric oxygen treatment + gentamicin

All the rats in the control group demonstrated normal renal morphology (score-0) [Fig. 1(a)]. All 28 intoxicated rats showed moderate to severe tubular degenerative, necrotic and regenerative changes. Group 1 had an average score of 3.4 (SD ±0.48), group 2 had an average score of 3.3 (SD ±0.6) and group 3 had an average score of 3.6 (SD ±0.26) without significant differences between the groups (*P = 0.6)* [Fig. 1 (b, c & d) respectively]. Details and comparison of pathological changes are illustrated in Fig. 2.

## Discussion

This study failed to prove the efficacy of HBOT in preventing or ameliorating gentamicin-induced renal failure, in a rat model. Biochemical parameters, gentamicin plasma levels and body weight were similar in all groups. However, two rats in the HBOT group died.

HBOT is considered a safe procedure. However, two rats developed sudden cyanosis and respiratory distress during the decompression phase of HBOT. The possible explanation for this phenomenon may be related to any relatively rapid decompression in the presence of trapped gas, whether oxygen, air or a gas mixture. During the decompression phase the trapped gas expands and may produce tension pneumothorax. This event is rare; one pneumothorax was reported in one study of 30 patients [10].

Previously, Solmazgul et al. [11] demonstrated that HBOT produced marked protection against ischemia of 30 min followed by 24-h reperfusion after the right nephrectomy in rats. These authors showed regression of the elevated plasma creatinine to normal concentrations in association with reversal of tubular damage and neutrophil infiltration by applying HBOT for 60 min, starting at the initial 15th min of reperfusion. However, the mechanisms underlying the beneficial effects of HBOT were not studied in this study. Other studies provided more information regarding the mechanisms underlying acute renal failure and support the use of HBOT for treatment of rats subjected to renal sepsis or ischemia/reperfusion injury [5, 6]. These studies demonstrated that HBOT induced renal vasodilatation and an increase in total renal blood. Moreover, they demonstrated that the improvement in renal function following HBOT occurs in parallel with a similar increase in the antioxidant/oxidant ratio in the ischemic kidney. This suggests that the latter mechanism in the HBOT group may contribute to the improvement in renal function in kidneys recovering from ischemic injury.

Oxidative stress is implicated in the pathogenesis of gentamicin-induced nephrotoxicity. The inhalation of oxygen that is greater than typical concentrations can stimulate ROS generation in several cell types. However, the results of studies are conflicting. Some studies of HBO treatments show that oxidative damage in several cell types increases [12, 13], while others conclude the opposite [14, 15]. HBOT has been used successfully to treat some renal medical conditions [16].However; there is evidence of renal impairment when HBOT is used in combination with nephrotoxic drugs. In one study, cisplatin caused more renal damage when HBOT was administered, but no renal pathological effects were observed with HBOT alone [7, 17]. The objective of another study that was performed at our medical center is to investigate the effect of HBOT on vancomycin-induced nephrotoxicity in rats. The results of this study showed that vancomycin-induced nephrotoxicity is actually worsened by HBOT treatment [8]. Similarly to gentamicin, oxidative stress might underlie the pathogenesis of vancomycin-induced nephrotoxicity, as suggested by animal studies [18, 19]. Another study performed by our group, showed alleviation of nephrotoxicity induced by amphotericin-B in rats treated with HBOT [9]. Vasoconstriction is the suggested mechanism of the amphotericin-B involved in inducing nephrotoxicity, thus increasing the amount of oxygen by using HBOT, which might be helpful in similar cases. On the other hand, HBOT may not have a nephroprotective role in toxicology cases of drugs that may have oxidative stress involved with nephrotoxicity such as vancomycin and gentamicin.

Pretreatment with HBOT was not evaluated in the present study. HBOT pretreatment has been used in patients before exposure to clinical situations with beneficial effects, but the mechanisms of action have not yet been ascertained. It has been postulated that during the reperfusion of ischemic tissue, oxygenated blood increases numbers and activities of oxidants generated in tissues. A previous report showed that HBOT preconditioning caused activation of anti-oxidative agents [20].

The current study revealed significantly higher serum levels of calcium in the group treated with a normal pressure supplement of oxygen. Adding pressure in the HBOT group did not have any effect on the plasma level of calcium. Experimental studies have demonstrated that gentamicin impaired calcium transport in the renal tubules resulting in a significant increase in urinary calcium excretion [21]. Our finding may suggest some renal benefit of oxygen supplements that was neutralized by adding pressure. This observation needs to be further investigated.

Regarding the plasma levels of gentamicin, no statistically significant differences were observed between the groups. This finding is in concordance with previous studies that examined the influence of HBOT on the pharmacokinetics of several drugs including gentamicin and revealed no changes [22, 23].

The pathological findings in the current study are compatible with the known pathological changes described previously in the literature. A central aspect of aminoglycoside nephrotoxicity is its tubular effect. Previous studies showed that treatment of experimental animals with gentamicin results in apoptosis [24], as well as necrosis [25] of tubular epithelial cells. Gentamicin cytotoxicity occurs in those cell types in which the drug accumulates. In the kidneys, these cells constitute of the epithelial cells in the cortex, mainly in the proximal tubule of experimental animals [26] and humans [27] and also in the distal and collecting ducts [28].

The limitations of this study include the lack of titrated HBOT or HBOT pretreatment or gentamicin dose, the short period of follow-up, the lack of analysis of free radicals and other kidney injury biomarkers. Nevertheless, the study’s significance lies in its testing, for the first time as far as we know, the effect of HBOT on gentamicin-induced renal failure.

## Conclusions

Our study evaluated the biochemical and the pathological efficacy of HBOT on acute renal failure induced by gentamicin. We found that treatment of gentamicin-induced nephrotoxicity with either HBOT or 100% oxygen for a consecutive 5 days had no beneficial renal effect, and mortality was observed only in the HBOT group.

## Abbreviations

ATA: Atmospheres absolute
CO2: Carbon dioxide
HBOT: Hyperbaric oxygen treatment
IACUC: Institutional animal care and use committee
